# Prevalence and antimicrobial susceptibility pattern of urinary tract infection among pregnant women attending Hargeisa Group Hospital, Hargeisa, Somaliland

**DOI:** 10.1038/s41598-022-05452-z

**Published:** 2022-01-26

**Authors:** Abdikhaliq Hussein Ali, Dawit Yihdego Reda, Moges Desta Ormago

**Affiliations:** Hawassa University College of Medicine and Health Science, Hawassa, Ethiopia

**Keywords:** Microbiology, Diseases

## Abstract

The aim of this study was to determine the prevalence, antimicrobial susceptibility pattern and associated factors of urinary tract infection (UTI) among pregnant women attending Hargeisa Group Hospital (HGH), Hargeisa, Somaliland. A cross-sectional study was conducted at HGH, Hargeisa, Somaliland and participants were selected by systematic random sampling technique. Clean catch midstream urine samples were collected from 422 participants and cultured and antimicrobial susceptibility pattern was determined for the isolates. Univariable and multivariable logistic regression analyses were utilized to identify the independent risk factors for UTI. The prevalence of UTI was 16.4% (95% CI 13.3–19.9). The predominant bacteria isolate was *E. coli* (43.5%) followed by *Coagulase negative staphylococcus (*CoNS*)* 11(16%), *S. aureus* 9(13%), *K. pneumonia* 6(8.7%), *Pseudomonas aeruginosa* 5(7.2%), *Proteus mirabilis* 4(5.8%), *Citrobacter spp* 3(4.4%) and *M. morganii* 1(1.5%) Gram negative bacilli were resistant to ampicillin (96%) and tetracycline (71.4%) and Gram-positive cocci were also resistant to ampicillin (90%), tetracycline (55%). Multidrug resistance was observed in 85.5% of bacterial isolated. No formal education participants, previous history of catheterization and previous history of UTI had 3.18, 3.22 and 3.73 times respectively more likely to develop UTI than their counterparts. Culture and susceptibility test is vital for appropriate management of UTI in the study area.

## Introduction

Urinary tract infection (UTI) is one of the highest frequent problem caused by some bacteria in a pregnant woman, which can lead to cause the significant complications for both mother and fetus^1^. The prevalence of the infection is higher among pregnant women than non-pregnant women and it is a major health problem reported among 20% of the pregnant women and a common cause of admission in obstetrical wards^2^.

If the infection is left untreated, it results in low birth weight fetus, intrauterine growth retardation, preterm labor and premature babies, intrauterine fetal death, and increased prenatal mortality and morbidity as well as maternal complications including anemia, preeclampsia, renal failure, septicemia, and adult respiratory syndrome^3^.

In Hargeisa Group Hospital, Hargeisa, Somaliland, routine culture and antimicrobial susceptibility testing of UTI are not performed and the treatment is on an empirical basis. This may promote the overuse of antibiotics and the development of resistant microbial strains. There was no published information on the prevalence of UTI and antimicrobial susceptibility pattern in Somaliland. Therefore, this study was carried out to determine the prevalence, antimicrobial susceptibility pattern and associated factors of urinary tract infection among pregnant women attending antenatal care at HGH, Hargeisa, Somaliland.

## Method

Cross-sectional study was conducted at antenatal care (ANC) in HGH, Hargeisa, Somaliland from May to October 2020. Being pregnant and having a follow up in the ANC clinic of HGH were included in the study. Pregnant women who received antibiotics within two weeks before ANC follow up were excluded. A structure questionnaire was used by trained nurses for the collection of Clinical and Socio-demographic data.

Ten milliliters of clean catch midstream urine sample were collected in a wide mouthed sterile container from each study participant. The collected urine sample was labeled and delivered to the hospital laboratory within one hour^4^.

The collected urine samples were processed and using a calibrated loop (0.001 ml) urine specimens were inoculated in to blood agar and MacConkey agar plates. After overnight incubation at 37 °C for 24–48 h’ colonies were counted to check significant growth. Colony counts of bacterial growth of > 10^5^/ml of urine were significant^5,6^. All positive cultures with significance bacteriuria were then identified at species level by their colony characteristics, Gram-staining reaction and by the pattern of biochemical profiles using the standard microbiological technique . The enterobacteriaceae were identified by H2S production and carbohydrate fermentation in KIA agar, indole production, citrate utilization, motility test, urease test and oxidase test. The Gram positive cocci were identified using catalase and coagulase tests^4^.

Kirby-Bauer disc diffusion method was used for the antibiotic susceptibility test. Three to five pure colonies were transferred into a tube containing 4–5 mL nutrient broth and mixed gently and then incubated at 35–37 °C for 2–6 h. The turbidity of the suspension was compared with McFarland 0.5 tubes to standardize the inoculums size^7^.

By using a sterile cotton swab dipping it into the suspension evenly over the entire surface of Mueller–Hinton agar (MHA) (Oxide Ltd, Hampshire, UK). The inoculated plates were left at room temperature to dry for 3–15 min. The following commercially available antibiotic discs were used with their respective concentrations: for Gram-negative bacilli augmentin, (AMC, 20/10 µg), ampicillin (AMP, 10 µg), ciprofloxacin (CIP, 5 µg), norfloxacin (NOR, 10 µg), trimethoprim + sulphamethazole (SXT, 25 µg, 1.25/23.75 µg), gentamicin (GEN, 10 µg), ceftriaxone (CRO, 30 µg), nalidixic acid (NA, 30 µg), meropenem (10 μg), tetracycline (TE; 30 μg) and nitrofurantoin (F, 300 µg) and for Gram-positive cocci erythromycin (ERY, 15 μg), penicillin (PEN, 10 µg), augmentin, (AMC, 20/10 µg), ampicillin (AMP, 10 µg), ciprofloxacin (CIP, 5 µg), trimethoprim + sulphamethazole (SXT, 25 µg,1.25/23.75 µg), gentamicin (GEN, 10 µg), ceftriaxone (CRO, 30 µg), nitrofurantoin (F, 300 µg), tetracycline (TE; 30 μg) and cefoxitin (CXT 30 µg) and finally, the result was reported as sensitive (S), intermediate (I) or resistance (R) by measuring the diameter of zone of inhibition or hemolysis^8^.

All filled questionnaires for this study was checked visually, coded and entered into excel and then exported to SPSS version25 software (SPSS Inc., Chicago, IL, USA) for analysis. Bivariate logistic regression was used to determine predictors of culture confirmed UTI. For those variables, which *P*-value < 0.25 in the bivariate, the analysis was further entered into the multivariable logistic regression model^9^. Associations between dependent and independent variables were assessed and its strength was described using odds ratios at 95% confidence intervals. A statistically significant association considered as *P*-value < 0.05.

Ethical approval was obtained from the institutional review board of Hawassa University, College of medicine and health sciences (Ref No: IRB/204/12). Informed consent was obtained from all participants. All methods were carried out in accordance with relevant guidelines and regulations.

### Ethics approval and consent to participate

Ethical approval was obtained from the institutional review board of Hawassa University, College of medicine and health sciences (Ref No: IRB/231/11). Informed written consent was obtained from all participants. All methods were carried out in accordance with relevant guidelines and regulations. Clinicians were communicated the findings of culture and sensitivity tests.

### Consent for publication

Individual data such as images and videos did not accompany this particular manuscript and hence consent for publication is not applicable.

## Results

A total of 422 pregnant women were participated during the study period. In this study the mean age of study participants was 30.9 (± 5.6 SD) years within the age range of 18–44 (Table 1).

**Table 1:** Sociodemographic characteristics of pregnant women attended antenatal care at HGH, Hargeisa, Somaliland, 2020 (n = 422).

Variables		Frequency	Percent (%)
Age ( in years)	< 19	17	4.0
	20–25	88	20.9
	26–30	119	28.2
	31–35	125	29.6
	36–40	40	9.5
	41–45	33	7.8
Residence	Urban	398	94.3
	Rural	24	5.7
Marital status	Widowed	8	1.9
	Married	407	96.4
	Divorced	2	0.5
	Separated	5	1.2
Educational status	No formal education	79	18.7
	Primary education (1–8)	185	43.8
	Secondary education(9–12)	112	26.5
	Higher education (> 12)	46	10.9
Family Income	< $100	77	18.2
	$101–200	191	45.3
	$201–300	116	27.5
	< $300	38	9.0
Occupational status	House wife	243	57.6
	Merchant	92	21.8
	Governmental employee	78	18.5
	Student	9	2.1

Obstetrics and clinical characteristics are indicated in Table 2.

**Table 2:** Obstetrics and clinical characteristics of pregnant women attended antenatal care at HGH, Hargeisa, Somaliland, 2020 (n = 422).

Variables		Frequency(n)	Percent (%)
Gestational period	First trimester	87	20.6
	Second trimester	137	32.5
	Third trimester	198	46.9
Gravida	Primigravida	56	13.3
	Multigravida	366	86.7
History of Catheterization	No	394	93.4
	Yes	28	6.6
History of diabetes mellitus	No	412	97.6
	Yes	10	2.4
History of abortion	No	405	96.0
	Yes	17	4.0
History of obstetric and gynecologic surgery	No	413	97.9
	Yes	9	2.1
History of premature labor	No	416	98.6
	Yes	6	1.4
History of previous UTI	No	370	87.7
	Yes	52	12.3

**Figure 1:**
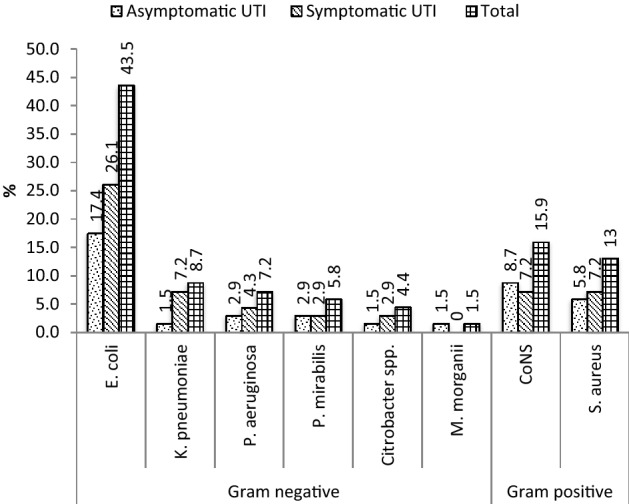
Bacterial profile isolated from urine culture of symptomatic and asymptomatic pregnant women with UTI attended antenatal care at HGH, Hargeisa, Somaliland, 2020. *CONS* coagulase-negative staphylococci, *UTI* urinary tract infection.

Overall prevalence of UTI were 16.4% (95% CI 13.3–19.9) of which 40(9.5%) was symptomatic UTI and 29(6.9%) was asymptomatic UTI. Of 69 positive cases, eight different types of bacteria were identified. The majority of the isolates belong to the Gram negative bacilli 49(71%). Among the isolates the predominant bacteria were *E. coli* 30(43.5%), followed by Coagulase negative staphylococcus (CoNS) 11(15.9%), *S. aureus* 9(13%), *K. pneumonia* 6(8.7%), *Pseudomonas aeruginosa* 5(7.2%), *Proteus mirabilis* 4(5.8%), *Citrobacter spp* 3(4.4%) and *M. morganii* 1(1.5%) (Fig. 1).

Gram negative bacilli were resistant to ampicillin (96%), tetracycline (71.4%), trimethoprim-sulfamethoxazole (57.1%), amoxicillin clavulanic acid (55.1%) and nalidixic acid (51%) and high rate of sensitive were also observed to meropenem (95.9%), ceftriaxone (79.6%), norfloxacin (77.5%), gentamicin (75.5%), nitrofurantoin (75.5%) and ciprofloxacin (71.4%). With regard to specific isolated Gram negative bacilli, Escherichia coli were highly resistant to ampicillin (93.3%), tetracycline (73.3%), trimethoprim–sulfamethoxazole (60%), nalidixic acid (53.3%) and amoxicillin clavulanic acid (46.7%) but sensitive to meropenem (96.7%), norfloxacin (90%), ceftriaxone (83.3%), nitrofurantoin (80%), gentamicin (73.3%) and ciprofloxacin (66.7%).

All *K. pneumonia* isolates showed resistant to ampicillin, and 66.7% were resistant to norfloxacin and amoxicillin clavulanic acid each and 50% were resistant to tetracycline, nalidixic acid and trimethoprim-sulfamethoxazole each but *K. pneumonia* was sensitive to meropenem (100%), gentamycin (83.3%) and 66.7% were sensitive to ceftriaxone, ciprofloxacin and nitrofurantoin each.

*P. aeruginosa* were resistant to ampicillin (80%), tetracycline (57.1%), trimethoprim- sulfamethoxazole (57.1%) while highly sensitive were observed to meopenem, gentamicin, norfloxacin and ciprofloxacin to 80% for each and 60% were sensitive to ceftriaxone and nitrofurantoin each. *Proteus mirabilis* were resistant to ampicillin (100%) and 75% to each of tetracycline and amoxicillin clavulanic acid while 100% sensitive to meropenem, gentamicin and ceftriaxone each and 75% sensitive to ciprofloxacin and norfloxacin each. *Citrobacter spp* were highly resistant to ampicillin (100%) and 66.7% were resistant to tetracycline, nalidixic acid, trimethoprim-sulfamethoxazole and amoxicillin clavulanic acid each but *Citrobacter spp* were highly sensitive to meropenem, ciprofloxacin and nitrofurantoin and 66.7% were sensitive to gentamicin, norfloxacin and ceftriaxone each.

*M. morganii* were resistant to ampicillin, tetracycline, nalidixic acid, trimethoprim-sulfamethoxazole, norfloxacin and amoxicillin clavulanic acid but all *M. morganii* were sensitive to meropenem, ceftriaxone, ciprofloxacin and nitrofurantoin (Table 3).

**Table 3:** Antimicrobial susceptibility pattern of gram-negative bacilli isolated from pregnant women with UTI attended antenatal care at HGH, Hargeisa, Somaliland, 2020 (n = 49).

Isolates	Pattern	Antibiotics (%)										
		AMP	CIP	CRO	AMC	SXT	NOR	GEN	NA	F	MER	TTC
E. coli(N = 30)	S	1(3.3)	20(66.7)	25(83.3)	14(46.7)	9(30)	27(90)	22(73.3)	13(43.3)	24(80)	29(96.7)	4(13.3)
	I	1(3.3)	2(6.7)		2 (6.7)	3(10)	1(3.3)	2(6.7)	1(3.3)	2 (6.7)		4(13.3)
	R	28(93.3)	8(26.7)	5(16.37)	14(46.7)	18(60)	2(6.7)	6(20)	16(53.3)	4(13.3)	1(3.3)	22(73.3)
K. pneumonia (N = 6)	S		4(66.7)	4(66.7)	1(16.7)	1(16.7)	2(33.3)	5(83.3)	3(50)	4(66.7)	6 (100)	1(16.7)
	I			2(33.3)	1(16.7)	2(33.3)						2(33.3)
	R	6 (100)	2(33.3)		4(66.7)	3(50)	4(66.7)	1(16.7)	3(50)	2(33.3)		3(50)
P. aeruginosa (N = 5)	S		4(80)	3(60)	2(40)	1(20)	4(80)	4(80)	2(40)	3(60)	4(80)	1(20)
	I					1(20)	1(20)		1(20)	1(20)	1(20)	
	R	5 (100)	1(20)	2(40)	3(60)	3(60)		1(20)	2(40)	1(20)		4(80)
P. mirabilis (N = 4)	S		3(75)	4(100)	1(25)	2(50)	3(75)	4(100)	2(50)	2(50)	4 (100)	1(25)
	I					1(25)			1(25)			
	R	4 (100)	1(25)		3(75)	1(25)	1(25)		1(25)	2(50)		3(75)
Citrobacter spp.(N = 3)	S		3(100)	2(66.7)	1(33.3)	1(33.3)	2(66.7)	2(66.7)	1(33.3)	3(100)	3(100)	
	I						1(33.3)					1(33.3)
	R	3 (100)		1(33.3)	2(66.7)	2(66.7)		1(33.3)	2(66.7)			2(66.7)
M. morganii (N = 1)	S		1(100)	1(100)						1(100)	1(100)	
	I							1(100)				
	R	1(100)			1(100)	1(100)	1(100)		1(100)			1(100)
Total(N = 49)	S	1(2)	35(71.4)	39(79.6)	19(38.8)	14(28.6)	38(77.5)	37(75.5)	21(42.9)	37(75.5)	47(95.9)	7(14.2)
	I	1(2)	2(4.1)	2(4.1)	3(6.1)	7(14.3)	3(6.1)	3(6.1)	3(6.1)	3(6.1)	1(2)	7(14.2)
	R	47(96)	12(24.5)	8(16.3)	27(55.1)	28(57.1)	8(16.3)	9(18.4)	25(51)	9(18.3)	1(2)	35(71.4)

*AMP* ampicillin, *CIP* ciprofloxacin, *CRO* ceftriaxone, *AMC* amoxicillin clavulanic acid, *SXT* trimethoprim–sulfamethoxazole, *NOR* norfloxacin, *GEN* gentamicin, *NA* nalidixic acid, *F* nitrofurantoin, *MER* meropenem, *TTC* tetracycline, *S*, *I*, *R* sensitive, intermediate, resistant.

The Gram-positive bacilli were resistant to ampicillin (90%), tetracycline (55%), trimethoprim-sulfamethoxazole (50%), and amoxicillin clavulanic acid (50%) while sensitive to erythromycin (85%), cefoxitin (85%), ceftriaxone (75%), nitrofurantoin (75%), gentamicin (70%) and ciprofloxacin (85%).

Coagulase negative staphylococci (CoNS) were highly resistant to ampicillin (81.8%), tetracycline (54.5%), and amoxicillin clavulanic acid (45.4%) but sensitive to erythromycin (81.8%), cefoxitin (81.8%), ceftriaxone (72.7%), nitrofurantoin (72.7%) and gentamicin (72.7%), ciprofloxacin (63.6%) and trimethoprim–sulfamethoxazole (54.5%).

*S. aureus* were highly resistant to ampicillin (100%) and 55.6% were resistant to tetracycline, trimethoprim- sulfamethoxazole and amoxicillin clavulanic acid each while sensitive to erythromycin (88.9%), cefoxitin (88.9%), ceftriaxone (77.8%) and nitrofurantoin (77.8%), ciprofloxacin (66.7%) and gentamicin (66.7%) (Table 4).

**Table 4:** Antimicrobial susceptibility pattern of gram-positive cocci isolated from pregnant women with UTI attended antenatal care at HGH, Hargeisa, Somaliland, 2020 (n = 20).

Isolates	Pattern	Antibiotics (%)										
		AMP	CIP	CRO	AMC	SXT	CXT	GEN	P	ERY	F	TTC
CoNS (N = 11)	S	1(9.1)	7(63.6)	8(72.7)	5(45.4)	6(54.5)	9(81.8)	8(72.7)	5(45.4)	9(81.8)	8(72.7)	3(27.3)
	I	1(9.1)	2(18.2)		1(9.1)			1(9.1)	2(18.2)	1(9.1)	1(9.1)	2(18.2)
	R	9(81.8)	2(18.2)	3(27.3)	5(45.4)	5(45.4)	2(18.2)	2(18.2)	4(36.4)	1(9.1)	2(18.2)	6(54.5)
*S. aureus* (N = 9)	S		6(66.7)	7(77.8)	1(11.1)	4(44.4)	8(88.9)	6(66.7)	4(44.4)	8(88.9)	7(77.8)	3(33.3)
	I		1(11.1)	1(11.1)	3(33.3)			2(22.2)	1(11.1)			1(11.1)
	R	9(100)	2(22.2)	1(11.1)	5(55.6)	5(55.6)	1(11.1)	1(11.1)	4(44.4)	1(11.1)	2(22.2)	5(55.6)
Total (N = 20)	S	1(5)	13(65)	15(75)	6(30)	10(50)	17(85)	14(70)	9(45)	17(85)	15(75)	6(30)
	I	1(5)	3(15)	1(5)	4(20)			3(15)	3(15)	1(5)	1(5)	3(15)
	R	18(90)	4(20)	4(20)	10(50)	10(50)	3(15)	3(15)	8(40)	2(10)	4(20)	11(55)

Among the total isolates (n = 69) multi drug resistance (MDR) was observed in 59 (85.5%) of bacteria isolated. In Gram-negative bacteria MDR were observed in 44/49 (89.8%) while gram-positive bacteria were observed in 15/20 (75%) respectively (Table 5).

**Table 5:** Multi drug resistance pattern of bacterial isolates from pregnant women with UTI attended antenatal care at HGH, Hargeisa, Somaliland, 2020 (n = 69).

Isolates	Frequency (%)							
	Total		R3	R4	R5	R6	≥ R7	MDR
**Gram-negative**	**49(71)**		**9(20.5)**	**10(22.7)**	**17(38.6)**	**4(9.1)**	**4(9.1)**	**44(89.8)**
*E. coli*	30(61.2)		7(77.8)	5(50)	11(64.8)	3(75)	1(25)	27(61.4)
*K. pneumonia*	6(12.2)		1(11.1)		2(11.8)		2(50)	5(11.4)
*P. aeruginosa*	5(10.2)			3(30)	2(11.8)			5(11.4)
*P. mirabilis*	4(8.2)		1(11.1)		1(5.8)		1(25)	3(6.8)
*Citrobacter spp.*	3(6.1)			2(20)	1(5.8)			3(6.8)
*M. morganii*	1(2.1)					1(25)		1(2.2)
**Gram-positive**	**20(29)**		**1(6.7)**	**8(53.3)**	**2(13.3)**	**3(20)**	**1(6.7)**	**15(75)**
CoNS	11(55)			7(87.5)	1(50)	1(33.3)		9(60.0)
*S. aureus*	9(45)		1(100)	1(12.5)	1(50)	2(66.7)	1(100)	6(40.0)
**Total**	**69(100)**		**10(16.9)**	**18(30.5)**	**19(32.2)**	**7(11.9)**	**5(8.5)**	**59(85.5)**

R2 = resistance to two drugs, R3 = resistance to three drugs, R4 = resistance to four drugs, R5 = resistance to five drugs, R6 = resistance to six drugs and ≥ R7 = resistance to seven and more drugs, MDR = resistance for three or more antibiotics.

In bivariate analysis, Age of respondents [COR = 2.600 95% CI 0.725–9.319], Educational status [COR = 2.780 95% CI 0.965–8.006], Family income [COR = 3.559 95% CI 0.978–12.954], History of catheterization [COR = 3.154, 95% CI 1.388–7.170], History of abortion [COR = 2.220, 95% CI 0.756–6.517] and History of previous UTI [COR = 3.31, 95% CI 1.740–6.300] were found to be significantly associated with UTI among pregnant women and were to be a candidate for multivariate logistic regression analysis.

The result of multivariate analysis revealed that pregnant women with no formal education were 3.2 more likely to have UTI than those with higher education (> grade 12) [ AOR = 3.183 95% CI 1.027–9.866], family income ≤ $100 was 5.2 times higher risk of having UTI than those with family income > $300 [AOR = 5.225 95% CI 1.270–21.500] , the odds of having UTI among pregnant women who have previously indwelling catheter were 3.2 times higher than the odds in pregnant women who have not previously indwelling catheter [AOR = 3.216, 95% CI 1.287–8.038], pregnant women who have previous history of UTI were 3.7 more likely to occur the UTI compared with pregnant women that have not the previous history of UTI [AOR = 3.734, 95% CI 1.855–7.515] (Table 6).

**Table 6:** Bivariate and multivariate analysis for the assessment of factors associated with UTI among pregnant women attending antenatal care at HGH, Hargeisa, Somaliland, 2020 (n = 422).

Variables		UTI		COR(95% CI)	*P* values	AOR(95% CI)	*P* values
		Yes (%)	No (%)				
Age ( in years)	≤ 19	7(41.2)	10(58.8)	2.600 (0.725–9.319)	0.142	2.446(0.609–9.818)	0.207
	20–25	17(19.3)	71(80.7)	0.889 (0.331–2.389)	0.816	0.903 (0.313–2.608)	0.851
	26–30	20(16.8)	99(83.2)	0.750 (0.286–1.966)	0.559	0.813 (0.285–2.316)	0.698
	31–35	18(14.4)	107(85.6)	0.625 (0.236–1.652)	0.343	0.606 (0.212–1.732)	0.350
	36–40	0(0)	40(100)	0.000 (0.000)	0.998	0.000 (0.000)	0.997
	41–45	7(21.2)	26(78.8)	I			
Residence	Urban	66(16.6)	332(83.4)	1.392[0.403–4.800]	0.601		
	Rural	3(12.5)	21(87.5)	I			
Marital status	Widowed	2(25)	6(75)	I			
	Married	66(16.2)	341(83.8)	0.581 [0.115–2.940]	0.511		
	Divorced	0(0)	2(100)	0.000 [0.000]	0.999		
	Separated	1(20)	4(80)	0.750 [0.050–11.311]	0.835		
Educational status	No formal education	20	59	2.780 [0.965–8.006]	0.058	3.183 [1.027–9.866]	0.045
	Primary education (1–8)	29	156	1.524 [0.555–4.183	0.413	1.624 [0.553–4.766]	0.378
	Secondary education (9–12)	15	97	1.268 [0.432–3.719]	0.665	1.767 [0.552–5.658]	0.338
	Higher Education (> 12)	5	41	I			
Family Income	≤ $100	18	59	3.559 [0.978–12.954]	0.054	5.225 [1.270–21.500]	0.022
	$101–200	32	159	2.348 [0.680–8.103]	0.177	2.940 [0.761–11.352]	0.118
	$201–300	16	100	1.867 [0.513–6.793]	0.344	2.839 [0.694–11.615]	0.147
	> $300	3	35	I			
Occupational status	House wife	47	196	0.839 [0.169–4.171]	0.830		
	Merchant	12	80	0.525[0.097–2.830]	0.453		
	Governmental employee	8	70	0.400 [0.071–2.264]	0.300		
	Student	2	7	I			
Gestational Period	1st trimester	16	71	I			
	2nd trimester	23	114	0.895 [0.443–1.809]	0.758		
	3rd trimester	30	168	0.792 [0.407–1.544]	0.494		
Gravida	Primigravida	12	44	I			
	Multigravida	57	309	0.676 [0.337–1.359]	0.272		
History of Catheterization	No	59	335	I			
	Yes	10	18	3.154 [1.388–7.170]	0. 006	3.216 [1.287–8.038]	0.012
History of diabetes mellitus	No	67	345	I			
	Yes	2	8	1.287 [0.267–6.196]	0.753		
History of abortion	No	64	341	I			
	Yes	5	12	2.220 [0.756–6.517]	0.147	2.183 [0.672–7.092]	0.194
History of obstetric and gynecologic surgery	No	67	346	I			
	Yes	2	7	1.475 [0.300–7.258]	0.632		
History of premature labor	No	68	348	I			
	Yes	1	5	1.024 [0.118–8.899]	0.983		
History of previous UTI	No	51	319	I			
	Yes	18	34	3.311 [1.740–6.300]	0.000	3.734 [1.855–7.515]	0.000

*I* reference, *AOD* adjusted odds ratio, *COR* crude odds ratio, *N* number, *UTI* Urinary tract infection.

## Discussion

The overall prevalence of UTI in pregnant women in this study was 16.4%. This is comparable to the prevalence of UTI reported in in Mwanza City, Tanzania 16.8%^10^, in Nairobi, Kenya 15.7%^11^, in Kano, Northern Nigeria 15.8%^12^ and in Bangalore, India 15%^13^.

Whereas a higher prevalence was reported in Ambo Central Ethiopia 18.7%^14^, in Derna City Libya 49.3%^15^, in Ismailia, Egypt 29%^16^, in Benin city, Nigeria 21%^17^, in Saudi Arabia 53.5%^18^, in Nepal 37.8%^19^ respectively. A lower prevalence was reported from Iran 13.1%^20^, Gondar Northwest Ethiopia 10.4%^21^, Korela India 13.4%^22^, Khartoum Sudan 14%^23^. This variation in prevalence might be due to across different studies from one country to another and among regions of the same country might be attributed to the difference in associated factors, sample size, social habits of the community, the standard of personal hygiene and education^24^.

The prevalence of UTI among symptomatic and asymptomatic pregnant women in this study was 9.5% and 6.9% respectively. The occurrence of UTI case among asymptomatic was in line with the previous study done in Cameroon 7.8%^25^, in Kanpur, India 7.3%^26^ and in Makkah, Saudi Arabia 8%^27^, On the other hand, a low prevalence of 0.13% In meta-analysis in Iran^28^, in Colombo, Sri Lanka 3.6%^29^ and in Ghana 5.5%^30^ was reported asymptomatic UTI. While higher prevalence was recorded in Hawassa, Southern 21.2%^31^, in Bangladesh 10.2%^32^ and in Nairobi, Kenya 21.5%^33^.

In this study the symptomatic study was 9.5%, These result of symptomatic UTI were agree with a study conducted from in Khartoum Sudan 12.1%^23^, in Makkah, Saudi Arabia 12%^27^ and in Northeastern Ethiopia 11.9%^34^. But higher prevalence rate in Mekelle Northern Ethiopia 21.1%^35^, in Bangladesh 17.9%^36^, in in Goba and Sinana Woredas, Bale Zone, Southeast Ethiopia 35.3%^37^ and in South-western Uganda 35%^38^. The differences may be the variation of methodologies and study populations might affect prevalence in different sites.

In this study, Gram-negative bacteria isolates were more prevalent (71%) than Gram-positive bacteria isolates (29%). A similar finding was found from Ambo town, Central Ethiopia 69.6% and 30.4% for Gram negative and Gram positive pathogens^14^, in Tanzania also Gram negative bacteria and Gram positive bacteria were reported 61.9% and 38.1%^39^ respectively. This could be due to the presence of unique structure in Gram negative bacteria which help for attachment to the uroepithelial cells and prevent bacteria from urinary lavage, allowing for multiplication and tissue invasion–resulting in invasive infection and pyelonephritis in pregnancy^40^.

Among isolated pathogens, were *E. coli* was the most predominant bacteria 43.5%, which is similar with previous studies in Ambo town, Central Ethiopia 46.4% of isolated cases^14^, in Bangalore, India 43.9%^41^ and in Nairobi, Kenya 40.0% was reported^42^. However, it was lower than reported in the previous studies conducted in different countries, which was India 53.8%, Italy 57.1% and Iran 57.25%^22,43,44^. *E. coli* is the most common microorganism in the vaginal and rectal area. Because of anatomical and functional changes and difficulty of maintaining personal hygiene during pregnancy, may increase the risk of acquiring UTI from *E. coli*^45^. The second most common isolate was CoNS 16% and comparable findings have been reported in different studies like in Karamara Hospital Jigjiga, Eastern Ethiopia 12%^46^, Saint Paul’s Hospital Millennium Medical College, Addis Ababa, Ethiopia 14.3%^47^ and in Dil Chora Referral Hospital, Dire Dawa, Eastern Ethiopia19.2%^48^.

In this study, susceptibility pattern of Gram-negative bacteria showed that most of the isolates were sensitive to meropenem (95.9%), ceftriaxone (79.6%), norfloxacin (77.5%), gentamicin (75.5%), nitrofurantoin (75.5%) and ciprofloxacin (71.4%) and comparable studies conducted in different study area like in Addis Ababa, Ethiopia that showed highly sensitive to meropenem (75.2%), nitrofurantoin (93.1%), gentamicin (85.2%), ceftriaxone (82.2%), cefuroxime (79.3%), and ciprofloxacin (75.2%)^47^ and in South Nigeria showed sensitive to gentamicin (53–100%), imipenam (67–93%), ciprofloxacin (between 57–75%)^49^, In Hawassa, Southern Ethiopia the present study, 80% of Gram negative bacteria were susceptible to meropenem, ciprofloxacin, gentamicin, nitrofurantoin, and norfoxacin^50^. In this study, the highest resistance was shown to ampicillin (93–100%) among gram-negative bacteria, this due to the drug is with low cost and often can be purchased without prescription in different areas. This implies that ampicillin cannot be used as empirical therapy for urinary tract infection particularly in the study area. This also agrees with the study done in Karamara Hospital Jigjiga, Eastern Ethiopia^46^.

In this study the other drugs also showed highly resistance to Gram-negative were tetracycline (71.4%), trimethoprim–sulfamethoxazole (57.1%), amoxicillin clavulanic acid (55.1%) and nalidixic acid (51%) and this agree the other study done in Dil Chora Referral Hospital, Dire Dawa, Eastern Ethiopia AMP (89.5%), amoxicillin (73.7%), and TTC (73.7%), NA (52.6%), except nitrofurantoin (57.9%)^48^ and in Mbarara Regional Referral Hospital, South-western Uganda were highly resistant to Amoxicillin, Ampicillin, and Amoxicillin/Clavulanic acid at 95.7%, 95.0%, and 72.9%^38^. The findings of this study is not in line with the reports from Kenya^42^. These differences could be due to variations in antibiotic prescription patterns across various countries.

In this current study, the Gram-negatives, the predominant isolate was *E. coli*, which is resistant to ampicillin (93.3%), TTC (73.3%), SXT (60%), nalidixic acid (53.3%) and AMC (46.7%). Similar findings have been reported from previous studies in Dire Dawa, Eastern Ethiopia, South-western Uganda and Addis Ababa, Ethiopia^38,47,48^. The other isolated Gram negative bacteria include *K. pneumonia* showed more than 65% sensitive to meropenem, gentamicin, ceftriaxone, ciprofloxacin and nitrofurantoin this agreed with the study done in South-western Uganda^38^, *K. pneumoniae* was 100% resistant to ampicillin, Similar findings were done in Adigrat General Hospital, Northern Ethiopia and Karamara Hospital Jigjiga, Eastern Ethiopia^46,51^.

In this study, the Gram positive bacterial isolates were relatively sensitive to erythromycin, cefoxitin, ceftriaxone, nitrofurantoin, gentamicin, ciprofloxacin and each accounted 85%, 85%, 75%, 75%, 70% and 65%. This was comparable with the finding from Ivory Coast, Dire Dawa, Eastern Ethiopia and Gonder Ethiopia^48,52,53^ However, in contrast with study report from Southern Ethiopia, which ceftriaxone was 100% resistant to gram-positive bacteria^54^.

In this study, Gram positive bacteria showed highly resistance to ampicillin 90% and tetracycline 55%. This could be due to the infrequent use of the drug in the study area. Comparable result was reported in Gonder Ethiopia^53^, in Lagos, Nigeria, and Benishangul Gumuz Region, Western Ethiopia^55^. Coagulase negative staphylococci, which were the predominant isolates from Gram-positives 55% and was found 63% to 81% sensitive to erythromycin, cefoxitin, ceftriaxone, nitrofurantoin gentamicin and ciprofloxacin. However, in contrast was shown nitrofurantoin to 26.7% resistance in study done in Ethiopia^56^, while comparable studies done in Hawassa, Ethiopia^50^.

In this study, *S. aureus* which constituted for 45% of the Gram positive bacteria showed 66.7–88.9% were sensitive to erythromycin, cefoxitin, ceftriaxone, nitrofurantoin, ciprofloxacin and gentamicin, this agree with study done in Hawassa, Ethiopia, Benishangul Gumuz Region, Western Ethiopia and Nairobi, Kenya^42,50,55^. In contrast to research done in Addis Ababa, Ethiopia, which erythromycin was highly resistant 60%^47^. However, this study showed 100% ampicillin to *S. aureus*, similar study done in Jigjiga, Ethiopia, Dire Dawa, Eastern Ethiopia and Addis Ababa, Ethiopia^46,48,57^. This is caused by use of empirical treatment against bacterial infections of the urinary tract infection in the study area. This implies that ampicillin cannot be used as empirical therapy for urinary tract infection particularly in the study area.

In this study, MDR was seen in 85.5% of all bacteria isolated. Our finding is higher than studies done in same regions of Ethiopia like 57.1% in Addis Ababa, and 73% in Mekelle^35,47^, in Tanzania 77%^39^ and in Eastern Uganda 77.5^58^. Our finding is lower than studies done in South-South Nigeria 100%^49^, in Kenya 96%^11^ and same regions in Ethiopia like in Dire Dawa 100%, Gondar 95% and Jigjiga 96%^21,46,48^. This indicates that multi drug resistance was found to be very high to the commonly used antibiotics. Antibiotic resistance has been recognized as the consequence of antibiotic use and abuse^59^. Therefore, the reasons for this alarming phenomenon might be inappropriate and incorrect administration of antimicrobial agents in empiric therapies and lack of appropriate infection control strategies, which can cause a shift to increase prevalence of resistant organisms in the community.

In the present study, the result of multivariable logistic regression models revealed that socio-demographic factors among pregnant women were statistically significance with no formal education and low level of family income (≤ $100) [*P* = 0.045, AOR = 3.183 (1.027, 9.866)] and [*P* = 0.022, AOR = 5.225 (1.270, 21.500)]. The non-formal education was agreed with study done in Goba and Sinana Woredas, Bale Zone, Southeast Ethiopia [AOR = 6.617; CI = 1.87–9.94]^37^. in contract with low level education the studies done in Medan, Indonesia, and Uyo, Nigeria^49,60^. Low-income status was another factor that was related with high prevalence of UTI among pregnant women. A similar finding was reported in other studies on pregnant women in Dire Dawa, Eastern Ethiopia and in Adigrat General Hospital, Northern Ethiopia^48,51^. This could be due to the relation of low socioeconomic status with nutrition and immunity especially in pregnant women. In contrast studies were done in Jigjiga, Ethiopia, Medan, Indonesia, and Northeastern Ethiopia^34,46,60^.

In the study, obstetrics and clinical characteristics were shown that, highly significant proportion of UTI was recorded among those study subjects with prior history of UTI. The multivariate logistic regression analysis of current study showed that 3.7 more likely to occur the UTI compared with pregnant women that have not the previous history of UTI [*P* = 0.000 AOR = 3.734 (1.855, 7.515)]. This finding is similar with report from Uganda (*P* = 0.002), Libya (*P* = 0.00), Egypt (*P* = 0.001), India (*P* = 0.0423), and same regions of Ethiopia like Gondar, (*P*-value = 0.001), Dire Dewa (*P*-value = 0.006) and Addis Ababa (*P* = 0.004)^15,21,22,38,47,48,61^. The possible explanation for this association could be due to the existence of antibiotic-resistant strains from the previous infection.

In the current study, participants with the previous history of indwelling catheterization had about 3.2 times chance of developing UTI [*P* = 0.012 AOR = 3.216 (1.287, 8.038)] among pregnant women. This finding agrees with similar reports from Northeastern Ethiopia, Addis Ababa, Ethiopia and Gonder Ethiopia^21,34,47^. This could be due to long duration of catheterization, frequent catheterization or contamination during inserting catheters. However other studies done in Dire Dawa, Eastern Ethiopia and Jigjiga Eastern Ethiopia disagreed of this study^46,48^.

In the present study, there was no statistical significant association between prevalence of UTI among pregnant women and maternal age, residence, marital status, occupation, gestational period, gravidity, History of diabetes mellitus, History of abortion History of obstetric and gynecologic surgery and History of premature labor. this results were agreed the report from Bangladesh^36^, Nairobi, Kenya except the maternal age^33^, Nigeria^49^, Goba and Sinana Ethiopia^37^, Dire Dawa, Eastern Ethiopia^48^ and Addis Ababa, Ethiopia except history of abortion^47^.

## Conclusion

The overall prevalence was 16.4%.The isolated bacteria were *E. coli, K. pneumonia, P. aeruginosa, P. mirabilis, Citrobacter spp., CoNS and S. aureus.*

. Majority of the isolates were resistant to the commonly prescribed antibiotics, therefore culture and antibiotic susceptibility testing was recommended before giving treatment to prevent antimicrobial resistance at least at Referral Hospital Setup and health information dissemination to the patients recommended to avoid self-medication practice.

## Data Availability

The datasets used and analyzed in the current study are available from the corresponding author on reasonable request.

## Abbreviations

ANC: Antenatal care
HGH: Hargeisa Group Hospital
MDR: Multi drug resistance
UTI: Urinary tract infection
